# A Report of Accelerated Coronary Artery Disease Associated with Cerebral Autosomal Dominant Arteriopathy with Subcortical Infarcts and Leukoencephalopathy

**DOI:** 10.1155/2015/167513

**Published:** 2015-09-07

**Authors:** Courtney B. Rubin, Virginia Hahn, Taisei Kobayashi, Andrew Litwack

**Affiliations:** ^1^Perelman School of Medicine at the University of Pennsylvania, Philadelphia, PA 19104, USA; ^2^Department of Medical Ethics and Health Policy, University of Pennsylvania, Philadelphia, PA 19104, USA; ^3^Department of Medicine, Perelman School of Medicine at the University of Pennsylvania, Philadelphia, PA 19104, USA; ^4^Division of Cardiology, Perelman School of Medicine at the University of Pennsylvania, Philadelphia, PA 19104, USA

## Abstract

Cerebral autosomal dominant arteriopathy with subcortical infarcts and leukoencephalopathy (CADASIL) is the most common heritable form of vascular dementia and it is caused by mutations in the *NOTCH3* gene. The neurologic manifestations of CADASIL syndrome have been well characterized; however, here we report one of the first *de novo* cases of CADASIL-associated coronary artery disease. A 45-year-old woman with a history of CADASIL and remote tobacco use presented with unstable angina. She was found to have diffuse and irregular narrowing of the left anterior descending artery and a drug eluting stent was deployed. Months later, she developed two subsequent episodes of unstable angina, requiring stent placement in the distal left anterior descending artery and the right coronary artery. Though the neurologic manifestations of CADASIL have been well described, these patients may also be predisposed to developing premature coronary artery disease. Patients with CADASIL and their physicians should be aware of this possible association because these patients may not be identified as high risk by traditional cardiovascular risk estimators. These patients may benefit from more aggressive interventions to reduce cardiac risk.

## 1. Introduction

Cerebral autosomal dominant arteriopathy with subcortical infarcts and leukoencephalopathy (CADASIL) is the most common heritable form of vascular dementia in adults and is caused by mutations in the* NOTCH3* gene [1]. Patients with CADASIL usually present with various central nervous system- (CNS-) related symptoms: migraine with aura, transient ischemic attacks, mood disturbances, apathy, and dementia [2]. The gold standard for diagnosis of CADASIL syndrome has been a skin biopsy showing the presence of pathognomonic granular osmiophilic material in arterial walls; however, follow-up* NOTCH3* mutation analysis is now available to confirm the diagnosis [3]. These patients also show characteristic white matter hyperintensities on magnetic resonance imaging. Thickening of the arterial wall ultimately leads to lumen stenosis and lacunar infarcts. Although histopathological involvement appears to be more diffuse, the clinical manifestations of CADASIL syndrome have been best characterized in the CNS. Here we report a case of CADASIL syndrome affecting the coronary vasculature.

## 2. Case Report

**Figure 1:**
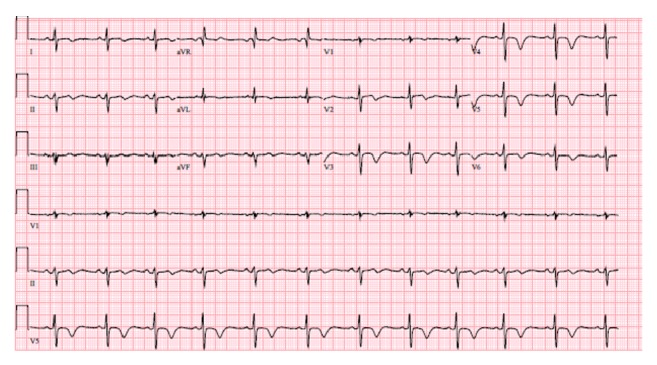
Electrocardiogram on the day of admission showing Wellens' waves.

**Figure 2:**
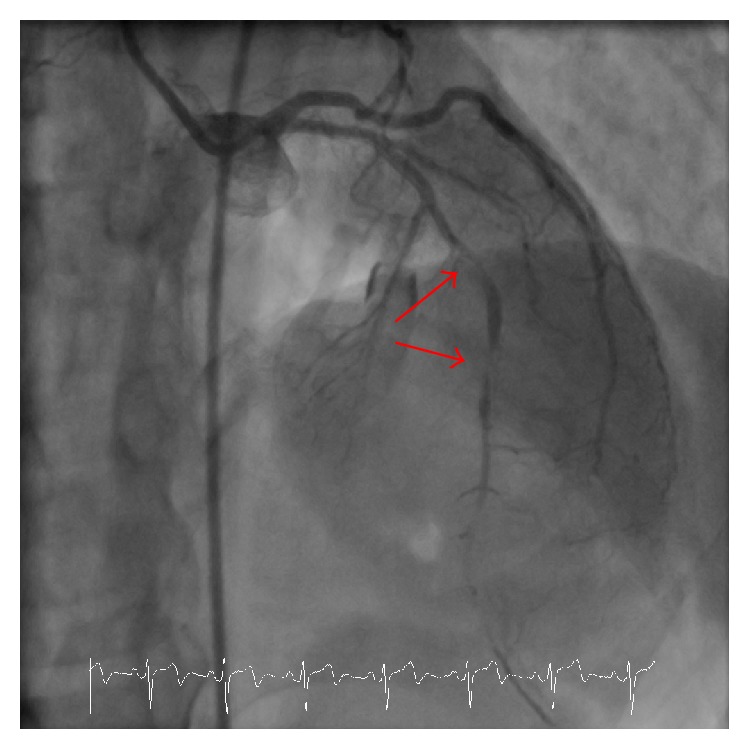
Initial coronary angiography showing two high grade lesions in series in the left anterior descending artery.

Patient KL is a 45-year-old premenopausal female with a history of CADASIL (diagnosed by* NOTCH3* mutation analysis) manifested by frequent transient ischemic attacks and chronic headaches who presented to the emergency department with unstable angina and new T wave inversions in the precordial leads, consistent with Wellens' T waves (Figure 1). KL underwent urgent coronary angiography, which showed diffuse and irregular narrowing of the left anterior descending artery, to which a drug eluting stent was deployed (Figure 2). After catheterization and stent deployment, KL was treated with aggressive risk reduction and pharmacotherapy with a high intensity statin, ACE inhibitor, aspirin, and clopidogrel. Despite these interventions, KL re-presented 6 months later with crescendo angina unrelieved with nitroglycerin. She underwent a repeat coronary angiogram, at which time a new significant stenosis was found in the right coronary artery, confirmed with a fractional flow reserve measurement. This lesion underwent drug eluting stent deployment.

Notably, KL does not have a history of hypertension, diabetes, hyperlipidemia, or a family history of coronary artery disease (Table 1). She has regular menstrual periods and is not on hormonal birth control. She has a 16-pack-year smoking history and quit 10 years ago. She has no family history of coronary artery disease or stroke. She has transient ischemic attacks every 1-2 months for the last 3 years and chronic headaches without aura on a daily basis for “as long as she can remember.”

## 3. Discussion

Thus far, CADASIL has been mainly characterized as a disease with primary CNS symptoms; however, limited data have suggested that the vasculopathy seen in CADASIL may affect other organ systems in addition to the CNS. One previous study has demonstrated the characteristic histopathologic findings along the arterial tree in muscle and skin biopsies from a patient with CADASIL syndrome [4]. Another study has shown that* NOTCH3* mutation carriers are at increased risk of early onset myocardial infarction compared to related nonmutation carriers [5]. This study employed the use of postmortem histology to demonstrate the presence of histopathologic findings consistent with CADASIL syndrome in the coronary microvasculature of one of the cases, further implicating CADASIL as an independent risk factor for early onset coronary disease. Although one criticism of this paper is that families with a history of CADASIL may be genetically predisposed to early coronary artery disease through a separate mechanism, the control patients in the study were related nonmutation carriers (who presumably carried the same genetic risks of early myocardial infarction) and despite this, the data continued to show a higher incidence of myocardial infarction in CADASIL carriers. Our report is the first to demonstrate premature coronary disease in a case of nonfamilial or* de novo* CADASIL and corroborates a growing evidence base demonstrating the cardiac sequelae of CADASIL.

This case demonstrates the possible acceleration of CAD due to KL's underlying CADASIL syndrome. First, the diffuse narrowing of the left anterior descending artery on coronary angiography is more indicative of a diffuse vascular process consistent with the pathogenesis of CADASIL. This pattern is in stark contrast to atherosclerotic plaque rupture, which usually results in a focal severe stenosis. Second, KL does not have any significant vascular risk factors such as diabetes, hypertension, dyslipidemia, early menopause, or family history of early CAD. Finally, KL's smoking history was felt not to be fully explanative of the aggressiveness and burden of her coronary disease based upon previous data showing that patients who have ceased smoking for 10 years carry a similar cardiovascular event risk as nonsmokers [6]. Thus at 45 years of age, she is at low risk for CAD using conventional risk assessment tools, suggesting a contribution of her CADASIL syndrome to her development of early CAD.

CADASIL patients frequently fall outside of current models for assessing cardiovascular risk, such as the Atherosclerotic Cardiovascular Disease (ASCVD) risk estimator. Additionally, many recommendations that guide cardiovascular risk management, such as the 2013 American College of Cardiology/American Heart Association cholesterol guidelines, assess traditional cardiac risk factors and do not take into account the contribution of systemic vasculopathies as in our patient. Failure to recognize the possible association of CAD with CADASIL may lead caregivers to misinterpret cardiac symptoms in patients with CADASIL and undertreat CAD.

The role and impact of aggressive reduction in modifiable cardiovascular risk factors such as hypertension, diabetes, obesity, and hyperlipidemia in patients with CADASIL are still unknown. However, this case report demonstrates that physicians who care for patients with CADASIL should frame common complaints of chest pain, exercise intolerance, or shortness of breath in the context of a higher pretest probability of early onset coronary artery disease.

## 4. Conclusions

Although the primary clinical manifestations of CADASIL syndrome mostly involve the CNS, we report a case of* de novo* CADASIL syndrome affecting the coronary vasculature and leading to multiple episodes of acute coronary syndrome. This case supports previously described pathophysiology of CADASIL-related coronary artery disease. This case report demonstrates that patients with CADASIL may have a higher probability of early onset coronary disease and may benefit from aggressive interventions to reduce risk.

## Figures and Tables

**Table 1:** Laboratory values and trends for KL's admissions for unstable angina.

	Admission 1	Admission 3
	(2/2014)	(8/2014)
Troponins	<.01 × 3	<.01 × 3
Total cholesterol	125	146
HDL	47	64
LDL	89	62
Triglycerides	90	102
A1C	5.1	NA
Blood pressure	144/90	127/74
